# Multi-modality cardiac imaging in the management of diabetic heart disease

**DOI:** 10.3389/fcvm.2022.1043711

**Published:** 2022-11-03

**Authors:** Malgorzata Wamil, Marcos Goncalves, Alexander Rutherford, Alessandra Borlotti, Patricia Ann Pellikka

**Affiliations:** ^1^Mayo Clinic Healthcare, London, United Kingdom; ^2^Deep Medicine, Oxford Martin School, Medical Science Division, Nuffield Department of Women’s and Reproductive Health, University of Oxford, Oxford, United Kingdom; ^3^Great Western Hospital NHS Trust, Swindon, United Kingdom; ^4^Perspectum Diagnostics, Oxford, United Kingdom; ^5^Department of Cardiovascular Medicine, Mayo Clinic, Rochester, MN, United States

**Keywords:** diabetic heart disease, diabetic cardiomyopathy, heart failure reduced ejection fraction (HFrEF), heart failure preserved ejection fraction (HFpEF), echocardiography, CT coronary angiography, cardiac magnetic resonance (CMR), MRI spectroscopy

## Abstract

Diabetic heart disease is a major healthcare problem. Patients with diabetes show an excess of death from cardiovascular causes, twice as high as the general population and those with diabetes type 1 and longer duration of the disease present with more severe cardiovascular complications. Premature coronary artery disease and heart failure are leading causes of morbidity and reduced life expectancy. Multimodality cardiac imaging, including echocardiography, cardiac computed tomography, nuclear medicine, and cardiac magnetic resonance play crucial role in the diagnosis and management of different pathologies included in the definition of diabetic heart disease. In this review we summarise the utility of multi-modality cardiac imaging in characterising ischaemic and non-ischaemic causes of diabetic heart disease and give an overview of the current clinical practice. We also describe emerging imaging techniques enabling early detection of coronary artery inflammation and the non-invasive characterisation of the atherosclerotic plaque disease. Furthermore, we discuss the role of MRI-derived techniques in studying altered myocardial metabolism linking diabetes with the development of diabetic cardiomyopathy. Finally, we discuss recent data regarding the use of artificial intelligence applied to large imaging databases and how those efforts can be utilised in the future in screening of patients with diabetes for early signs of disease.

## Introduction

World Health Organisation estimates that there are 422 million people living with diabetes mellitus worldwide and 1.5 million deaths are attributed to diabetes annually (1). Patients with diabetes are 2–3 times more likely to have heart disease and 84% of people over 65 with diabetes die from heart disease and stroke. Diabetes is associated with a significant shortening of life. On average, a 50-year-old with diabetes but no history of vascular disease dies approximately 6 years younger than a counterpart without diabetes (2). Early detection of the effect of diabetes on the cardiovascular system could improve preventive measures and initiation of treatments with proven cardiovascular benefit. In this review, we describe the roles of multimodality cardiac imaging in detecting the impact of diabetes on the myocardium and its use for longitudinal follow up.

### Definition of diabetic heart disease

Global prevalence of diabetes mellitus has been increasing rapidly over the last decade. This has given rise to the worldwide epidemic of diabetic cardiomyopathy, a condition covering the spectrum of myocardial abnormalities linked to the underlying metabolic disturbances observed in patients with diabetes in the absence of coronary artery disease. Numerous studies have explored pathophysiological mechanisms underlying changes in the cardiac structure and resulting function leading to the development of heart failure because of diabetes. In type 1 diabetes as compared to type 2 diabetes, the cardiovascular mortality remains similar, however, the impact of hyperglycaemia on the risk of development of heart disease is more pronounced in type 1 diabetes (3). The aetiology of so-called diabetic cardiomyopathy remains unknown but is likely multifactorial and therefore more difficult to characterise by a single modality. Multi-modality imaging is particularly useful in monitoring disease progression and evaluating the effectiveness of medical interventions. It remains debatable if cardiac imaging could be also used to screen asymptomatic patients with diabetes for the presence for early signs of heart disease.

Patients with diabetes, however, often present with accelerated atherosclerosis, which may lead to the development of ischaemic heart disease and subsequently heart failure reduced ejection fraction. Thus, it is frequently not possible to decipher the contribution of ischaemic and non-ischaemic factors to the development of diabetic heart failure.

### The risk of coronary artery disease in diabetes

Diabetes is associated with at least 2-fold increased risk of coronary artery disease and for many years has been regarded as ‘coronary risk equivalent’ (4). More recently, in recognition of the heterogeneity of patients with diabetes, clinical guidelines have suggested that further risk stratification is warranted when treatment decisions are considered (5, 6). Patients with diabetes and cardiovascular disease (CVD) or diabetes with target organ damage and those with three or more risk factors, as well as with the duration of diabetes more than 20 years are at very high risk (6). Most other subgroups are at moderate risk of developing coronary artery disease (CAD). In a systemic review and meta-analysis patient with diabetes without previous myocardial infarction (MI) had 43% lower risk of CAD than those without diabetes with established history of CAD (7).

### Multi-modality approach to imaging diabetic heart disease

Multimodality cardiac imaging, including echocardiography, cardiac computed tomography (CCT), cardiac magnetic resonance (CMR) and nuclear cardiology, has advanced our understanding and treatment of different pathologies included in the definition of diabetic heart disease. Non-invasive assessment of coronary artery disease, structural heart disease, arrhythmias, and heart failure, guides clinical management and leads to a significant improvement in patient outcomes. Multiple imaging modalities are used to detect the signs of diabetic heart disease and the full assessment frequently involves more than one type of scan.

There has been also an increased interest in using hybrid and fusion modalities combining two imaging techniques within one scan and incorporating machine learning into the analysis of images with the goal to improve earlier detection of metabolic and structural abnormalities which could lead to an improvement in long-term clinical outcomes.

## The role of echocardiography in the diagnosis of diabetic cardiomyopathy

Echocardiography plays an important role in the detection of subclinical dysfunction. Left ventricular dysfunction in patients with diabetes can have various presentations including predominantly systolic, diastolic, or mixed phenotypes (Table 1). Therefore, cardiac imaging should not only detect those abnormalities but also characterise the pathogenesis and offer deep phenotyping.

**TABLE 1:** Summary of studies assessing the role of echocardiography in the diagnosis of diabetic cardiomyopathy.

Authors	Year	Country	Age range (years)	Number of cases	Gender	Main findings	Relevant echocardiography parameters
Kwong et al. (73)		China	40–71	58	M and F	LV dyssynchrony and reduced myocardial perfusion in T2DM patients by RT-3DE and MCE assessment.	RT-3DE, MCE, MP, 3D EDV, 3D ESV, 3D EF, mechanical dyssynchrony assessment.
Schelbert et al. (74)		China	45–65	327	M and F	Myocardial perfusion abnormalities were identified in asymptomatic T2DM patients by MCE analysis.	MCE, IVST, PWT, IVSd, LVIDd, LVPWd, LVEF, transmitral inflow velocities, E/A ratio, DT, IVRT, LAVI.
Weber et al. (14)		Brazil	20–50	40	M and F	Reduced echocardiographic indices of diastolic function in T1DM patients noted compared to the control group.	LV GLS, biplane EF, IVSd, LVIDd, LVPWd, LVMI, Transmitral inflow E and A velocities, E/A ratio, DT, IVRT, TDI septal E’ and lateral e’ vel, Average E/e’ratio, LAVI. TAPSE, RV S’, RV GLS, RV FAC.
Rorth et al. (22)		Denmark	18–71	960	M and F	E/e’ were associated with increased risk of MACE and all-cause mortality in patients with T1DM.	LV GLS, LVEF, IVSd, LVIDd, LVPWd, LVMI, transmitral inflow E and A velocities, E/A ratio, TDI septal E’ and lateral e’ vel, average E/e’ratio, LAVI.
Ng et al. (75)		Denmark	39–60	1,093	M and F	Association between E/e’ and MACE in individuals with T1DM and without known heart disease.	LV GLS, LVEF, transmitral inflow E vel, TDI septal E’ and lateral e’ vel, average E/e’ratio.
Jellis et al. (76)		Egypt	34–71	60	M and F	Early detection and evaluation of systolic and diastolic dysfunction in T2DM, is superior when using strain and SR by TDI, compared with conventional doppler analysis.	LV longitudinal strain and strain rate derived from tissue doppler imaging.
Khan et al. (77)		Australia	>65	>150,000	M and F	LVH, diastolic dysfunction, fibrosis, reduced cardiac functional is observed in patients with diabetic cardiomyopathy.	LV GLS, LV GCS, LVM, LVM index to height, RWT, IVSd, LVIDd, LVPWd LVEF, transmitral inflow E and A vel, E/A ratio, TDI septal E’ and lateral e’ vel, Lat E/e’ratio, septal E/e’ ratio, average E/e’ratio, TDI septal and lateral S’, CFVR, LAVI, Svi.
Jorgensen et al. (21)		Denmark	57–74	933	M and F	E/e’ and GLS are echocardiographic T2DM gender specific parameters.	LV GLS, LV GLS rate, LV GCS, LV GCS rate, LVEF, IVSd, LVIDd, LVPWd, LVMI, transmitral inflow E and A velocities, E/A ratio, TDI septal E’ and lateral e’ vel, average E/e’ratio, LAVI.
Levelt et al. (78)		Iraq	35–47	151	M and F	There is a direct relationship between pre-clinical and clinical diastolic dysfunction and duration of diabetes.	Transmitral inflow E and A velocities, E/A ratio, TDI septal E’ and lateral e’ vel, Lat E/e’ ratio, average E/e’ratio, LAVI, TRPV.
Kristensen et al. (12)		Denmark	60–79	745	M and F	E/e’ ratio, transmitral doppler E-wave velocity, left ventricular mass and left atrial area were higher in patients with diabetes mellitus.	LVM, LVEDV, LVESV, IVSd, LVIDd, LVPWd LVEF, transmitral inflow E and A velocities, E/A ratio, TDI septal E’ and lateral e’ vel, Lat E/e’ ratio, average E/e’ratio, LAVI, LA area.
Rijzewijk et al. (79)		France	48–68	842	M and F	Association of echocardiographic variables with 3 different T2DM phenotypes.	LVMi, LVEDV, LVEDV index, LVESV index, IVSd, LVIDd, LVPWd LVEF, transmitral inflow E and A velocities, E/A ratio, TDI septal E’ and lateral e’ vel, Lat E/e’ ratio, average E/e’ratio, LAVI, LA area.
Ng et al. (80)		Australia	–	1,495	M and F	Follow-up recommended for patients with diabetes despite the low MACE rate in negative stress echocardiograms.	WMA, WMA at peak stress.
Hammer et al. (81)		Denmark	55–74	1,030	M and F	Echocardiographic abnormalities are very common in patients with T2DM. Echocardiography assessment should be considered in most patients regardless of	LVM index to height, LVEDV, LVEDV index, LVESV index, IVSd, LVIDd, LVPWd LVEF, transmitral inflow E and A velocities, E/A ratio, TDI septal E’ and
						cardiac symptoms and clinical characteristics.	lateral e’ vel, Lat E/e’ ratio, average E/e’ratio, LAVI, RV TAPSE.
Szczepaniak et al. (82)		Denmark	33–69	1,093	M and F	Myocardial function impairment in T1DM patients can be detected many years prior to the development of HF.	LV GLS, LVEF, transmitral inflow E vel. TDI septal e’ and lateral e’ and a’ vel. TDI e’/a’ and E/e’ratio. TDI Lateral S’ and Septal S’ vel.
Enomoto et al. (16)		Japan	36–71	77	M and F	Subclinical LV dysfunction defined as impaired longitudinal shortening is associated with diabetic microangiopathy and its accumulated effects.	2D LV GLS, 3D LV GLS, LV GSC, 3D EF, 3D EDV, 3D ESV. LVMI, IVSd, LVIDd, LVPWd, transmitral inflow E and A velocities, E/A ratio, TDI septal E’ and lateral e’ vel, Lat E/e’ ratio.
van der Meer et al. (83)		Australia	–	230	M and F	Subclinical LV dysfunction can be identified by GLS imaging and is associated with adverse outcome.	2D LV GLS.
Neubauer (84)		Italy	50–73	5,456	M and F	A normal stress echocardiography result is associated with a worse outcome for diabetic patients compared to patients without diabetes.	Resting WMA, peak WMA, resting WMSI, peak WMSI.

Echocardiography is often the first-line evaluation of cardiac structure and function given its high temporal and spatial resolution, safety, availability, and cost-effectiveness. Left ventricular volumes and ejection fraction derived from 3D echocardiography has shown accuracy and reproducibility comparable to cardiac MRI (8). Advancements in image-based analysis of local myocardial deformation, including strain and Doppler tissue imaging, have allowed for the quantitative assessment of subclinical myocardial dysfunction and myocardial strain (9–11). Echocardiography plays central role in the assessment of diastolic dysfunction so prevalent among people with diabetes and obesity.

### Diastolic dysfunction associated with diabetes

Key parameters assessing diastolic dysfunction and structural changes have been positively correlated with progression to clinical heart failure. Structural changes identified include larger end-systolic and end-diastolic dimension, greater left ventricular mass in the context of similar wall thickness and greater left atrial area (12). Several studies have assessed signs of pre-clinical diastolic dysfunction in patients with diabetes with consistent results (12–16). In the I-Preserve trial (Irbesartan in Heart Failure with Preserved Ejection Fraction) patients with diabetes were shown to have significant echocardiographic abnormalities associated with diastolic dysfunction comparing to non-diabetic counterparts (12). This suggests that the pathogenesis of diabetic cardiomyopathy at the early stages includes higher left atrial pressures, increased left ventricular stiffness with reduced myocardial relaxation and impaired left ventricular filling. Interestingly, prior to the onset of overt cardiac disease the degree of diabetic control, microvascular complication or insulin requirement is not associated with indices of heart function or reflectivity in type 2 diabetic patients (13). Noteworthy, the duration of diabetes has been associated with the progression to left ventricular diastolic dysfunction with early diastolic impairment noted in the first five years from diagnosis (13). Conversely, in type 1 diabetic patients, e’ mean has been identified to correlate with HbA1c values and duration of DM1 (14).

Hence those echocardiographic features of diastolic dysfunction as well as left ventricular hypertrophy are often regarded as early signs that diabetes affected the myocardium (17). Age, obesity, and hypertension can frequently affect those parameters of diastolic dysfunction. Obesity frequently coexists with type 2 diabetes and has an additive detrimental effect on diastolic function (18). Although diastolic dysfunction has been linked to such processes as myocardial fibrosis, myocardial triglyceride accumulation and insulin resistance, echocardiographic features of diastolic dysfunction have been also attributable to coronary microvascular dysfunction (19).

### Systolic dysfunction in patients with diabetes

Patients with history of diabetes and significant coronary artery disease commonly present with heart failure reduced ejection fraction (HFrEF). Echocardiograms of those patients may detect regional wall motion abnormalities, alterations in strain and in the later stage also reduced ejection fraction. Abnormal global longitudinal strain (GLS) has been described as an early sign of developing HFrEF in patients with diabetes (13, 20). The clinical implication of these changes has been further examined with the endpoint of increased admissions secondary to cardiovascular disease or death related to cardiovascular disease. Jøgensen et al. found that when assessing a broader range of patients with diabetes and clinical heart failure, the structural and functional changes were associated with higher risk of progression to the cardiovascular disease (21). LVEF, GLS and GLS rate, E/e’ ratio was associated with the progression to heart failure (21). E/e’ ratio in combination with NT-proBNP levels have been shown to identify patients at highest risk of cardiovascular disease (22). Another study found that in the community-based patients with diabetes, screening of asymptomatic patients without previous history of coronary artery disease resulted in better long-term outcome although the result could not be explained by the revascularisation alone (23).

### The role of stress echocardiography

Stress echocardiography has been shown to be an accurate and reliable imaging modality to diagnose coronary artery disease and predict the long-term outcome in patients with diabetes (24). Exercise stress echocardiography is often used to confirm diminished systolic and diastolic functional reserve in this population (25). Although the negative predictive value of exercise stress echocardiography for myocardial ischaemia is reduced in patients with diabetes when compared to the non-diabetic population, recent analysis using dual-imaging dipyridamole stress echocardiography combining conventional wall motion analysis with Doppler-derived coronary flow velocity reserve (CFVR) of the left anterior descending coronary artery showed that abnormal findings were strong and independent predictors of major cardiovascular events in patients with DM (26).

## The role of coronary CT in the management of coronary artery disease in people with diabetes

### Coronary artery calcium

It has been shown previously that patients with diabetes have higher burden of plaque disease than those without diabetes (27) and the extent of CAD in patients with diabetes is comparable to patients with previous MIs (28). Noteworthy, women with diabetes have similar extent of CAD to men, which is different than in the general population (28). Moreover, women with diabetes are at higher risk of MI then men despite low burden of CAD. High coronary artery calcium (CAC) score correlates with a higher number of cardiovascular events and an increased mortality (29–32). CAC is recognised as a better predictor of coronary artery events than the Framingham score and the UKPDS Risk Engine and has been shown to have much higher annual increase than in patients without diabetes. CAC has been also shown to be a good biomarker of CAD progression in people with diabetes (33–36) and therefore has been proposed for monitoring of asymptomatic patient with diabetes and CAD.

### CT coronary angiography

**FIGURE 1:**
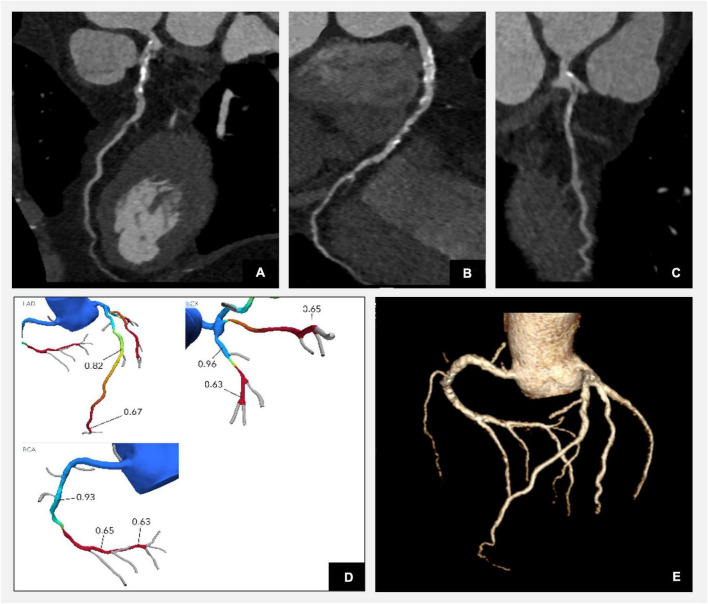
Illustration of corresponding anatomical coronary CTA plaque analysis and functional assessment with FFR-CT in a patient with diabetes. Reconstructions confirm three vessel disease with diffuse mixed plaque and severe flow limiting stenoses in the distal LAD, distal RCA, and proximal intermediate branch as per FFR-CT functional analysis. **(A)** CTA curved multiplanar reconstruction of the left anterior descending artery (LAD) demonstrating diffuse calcific plaque disease in the proximal segment with mild stenosis and low attenuation plaque in the mid vessel with severe moderate flow limiting stenosis as per angiographic assessment. **(B)** CTA curved multiplanar reconstruction of the right coronary artery (RCA) demonstrating diffuse mixed plaque disease throughout the vessel with most significant stenosis in the mid segment. **(C)** CTA curved multiplanar reconstruction of the intermediate artery reconstruction demonstrating diffuse mixed plaque disease with severe stenosis at the ostium of the vessel. **(D)** Computational fractional flow reserve (FFR) CT by HeartFlow indicating haemodynamically moderate flow impairment (FFR-CT of 0.82) across the lesion in the mid segment and severe (FFR-CT of 0.67) in the distal segment of the LAD, severe stenoses in the proximal intermediate (FFR-CT of 0.65) and distal segment of RCA (FFR-CT of 0.65). **(E)** Three-dimensional model illustrating the reconstruction of coronary arteries (Syngovia Siemens software).

Advancements in the CT technology resulting in a reduction of the radiation doses increased the popularity of CT coronary angiography (CTA) in the assessment of CAD in patients with diabetes (Figure 1). In asymptomatic patients with diabetes, on average studies reported the prevalence of obstructive coronary artery disease in 25–30% of patients, any coronary atheroma in 76% and multivessel coronary atheroma in 55% (37–39). In a cohort of asymptomatic patients with diabetes approximately 17% had multivessel disease and significant lesions in the left main stem or proximal left anterior descending artery (40). Moreover, an increased severity of coronary artery disease was associated with poor prognosis implying an important prognostic role of CTA in the management of patients with diabetes. Those results highlighted the role of CTA in finding high-risk patients even among asymptomatic patients. The FACTOR-64 (For Asymptomatic Obstructive Coronary Artery Disease Among High-Risk Diabetic Patients Using CT Angiography) randomised controlled trial evaluated the utility of routine CTA screening in patients with diabetes (41). CTA showed no atheroma in 31%, mild stenosis in 46%, moderate in 12%, and severe in 11% of patients. Although coronary CTA screening prompted aggressive risk factor modifications in 70% of patients, there was no significant reduction in CAD events in the group randomised to the CTA screening (41). Thus, CTA is currently not recommended to risk stratify patients with diabetes (42). An ongoing Computed Tomography Coronary Angiography for the Prevention of Myocardial Infarction (SCOT-Heart 2) randomised controlled trial investigates the role of CTA in a broader primary prevention population including patients with diabetes.

Myocardial computed tomography perfusion (CTP) imaging is an emerging CT-based method for detecting myocardial ischaemia with good accuracy (43) and correlated with nuclear myocardial perfusion imaging. The combination of anatomical information from CTA with functional assessment from CT adenosine-stress perfusion CT increases significantly the radiation exposure to the patients and therefore limits its wider use.

FFR-CT is an alternative functional technique, which combines anatomic and physiologic information in a single non-invasive test. Patients with diabetes have a high burden of coronary artery disease and it has been shown that every sixth asymptomatic patient at the time of the diagnosis of diabetes has haemodynamically significant plaque disease as evaluated by FFR-CT (44) (see an example of FFR-CT assessment in Figure 1). FFR-CT has improved specificity without impacting on the sensitivity of CTA in detecting haemodynamic significant coronary artery disease (45). Quality of image acquisition in CTA has long been established as important with multiple factors impacting calculation of FFR (46). Currently only HeartFlow technology is licenced by both the National Institute of Clinical Excellence and the US Food and Drug Administration for FFR-CT assessment (46, 47). Post-acquisition image processing occurs following images being sent to HeartFlow. Thus, producing FFR-CT results takes time with currently only >50% of images being processed in less than 5 h, limiting its use in the acute clinical setting. Local FFR-CT techniques will likely progress over time to improve streamlining, access, and timeliness of the technology.

European and American societies have recently introduced the use of CTA in patients with diabetes who present with a history of chest pain (6, 48). The SCOT-Heart trial, which recruited 444 patients with diabetes showed a significant reduction in the risk of cardiovascular mortality and myocardial infarction with CTA-guided management (49). The Prospective Multicentre Imaging Study for Evaluation of Chest Pain (PROMISE) randomised patients to CTA or stress test and showed that abnormal stress test was more specific for predicting primary outcome than CTA (50). Noteworthy, in the CTA arm 84% of events occurred in patients with calcium score above zero and only 43% of events in the functional test group. However, in the prespecified sub-analysis of 1908 recruited patients with diabetes, those who underwent CTA had a lower risk of cardiovascular event comparing to the stress test arm. The addition of computational fractional flow reserve may therefore improve even further identification of haemodynamically significant lesion (51). ISCHEMIA trial tested whether patients with moderate or severe ischaemia on stress test benefit from revascularisation in the setting of optimal medical therapy. There was no difference in the primary end point among participants with diabetes (41.8%) highlighting the importance of the optimal medical therapy (52).

### Peri-coronary adipose tissue attenuation on CT coronary angiography

**FIGURE 2:**
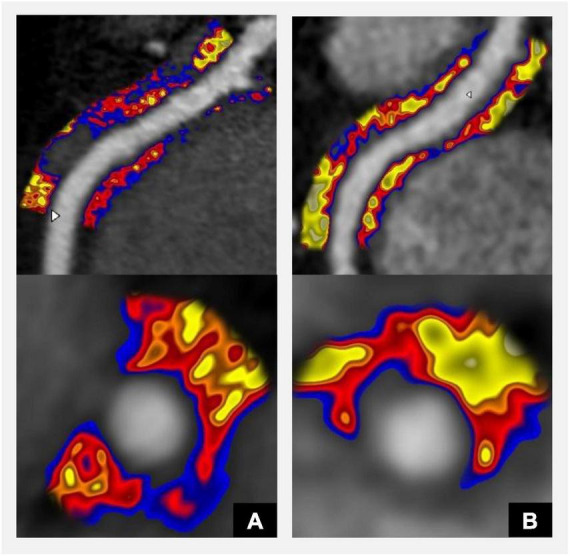
CT coronary angiography derived Fat Attenuation Index (FAI) derived from the analysis of Perivascular Adipose Tissue (PVAT) surrounding coronary arteries detects inflammation and may be used to guide the management of patients with diabetes and increased cardiovascular risk. Red-blue colour indicates inflammation and correlates with high risk of future cardiovascular events; yellow colour indicates low inflammation (55). FAI score can be provided for each of the main coronary arteries and considers FAI weighted for tube voltage, anatomical factors, and basic demographics. **(A)** Significantly increased coronary artery inflammation (FAI score 95th centile). **(B)** Follow-up scan after initiation of statin treatment shows significant reduction in inflammation (FAI score 58th centile). Panels **(A,B)** show CPR **(top panel)** and axial views **(bottom panel)**.

Mapping of peri-coronary adipose tissue attenuation (PCAT) on CTA has been recently proposed as a non-invasive marker of coronary artery inflammation (53). Several clinical studies have confirmed that so called fat attenuation index (FAI) can improve the prediction of coronary events beyond traditional risk factors and CTA metrics (53, 54). FAI was first derived from studies demonstrating that inflammatory markers released from inflamed coronary arteries modify the composition of perivascular adipose tissue. Those changes can be detected using a CTA. Thus, using FAI assessment we can extract additional information from a standard CTA scan relating to coronary artery inflammation (55). Noteworthy, it has been demonstrated that lesion-specific FAI enhances the predictive ability of CTA plaque characterisation for ischaemia as assessed by invasive coronary angiography (56). When FAI was added to the CTA stenosis estimation the AUC of such model was comparable to the one achieved with the invasive assessment of stenosis severity. The value of PCAT has been now also assessed as a predictive tool in patients with diabetes (57). Adding PCAT along with findings of CTA improved the model fit for predicting cardiovascular events in type 2 diabetes patients and helped to identify patients at a higher risk independently of the plaque characteristics. PCAT is a dynamic marker reported to be markedly reduced in response to anti-inflammatory treatment (58) (Figure 2).

## Nuclear medicine as imaging of choice in managing patients with diabetes

Stress myocardial perfusion scintigraphy (MPS) is widely used in patients with and without diabetes to detect haemodynamically significant coronary artery disease. It has been reported that in 20–25% of asymptomatic patients with the diagnosis of diabetes MPS detects ischaemia (59, 60) and in those patients it is associated with cardiovascular events (61). Microvascular/endothelial dysfunction is frequently described in patients with diabetes and can be assessed by quantitative positron emission tomography (PET). Measurements with a blood flow radiotracer such as ^82^Rubidium, ^13^N-ammonia or ^15^O-water at rest and after vasodilator-stress enable calculation of coronary flow reserve (62). Moreover, PET has been also used to study utilisation of glucose and fatty acids in 31 young women and showed that insulin resistance correlated with utilisation and oxidation of fatty acids (63). Due to the complexity of its protocol and the high price, it remains to be a research tool rather than a technique which could be used to assess patients.

## The role of cardiac magnetic resonance in the detection of aberrant myocardial metabolism and cardiac structure

**FIGURE 3:**
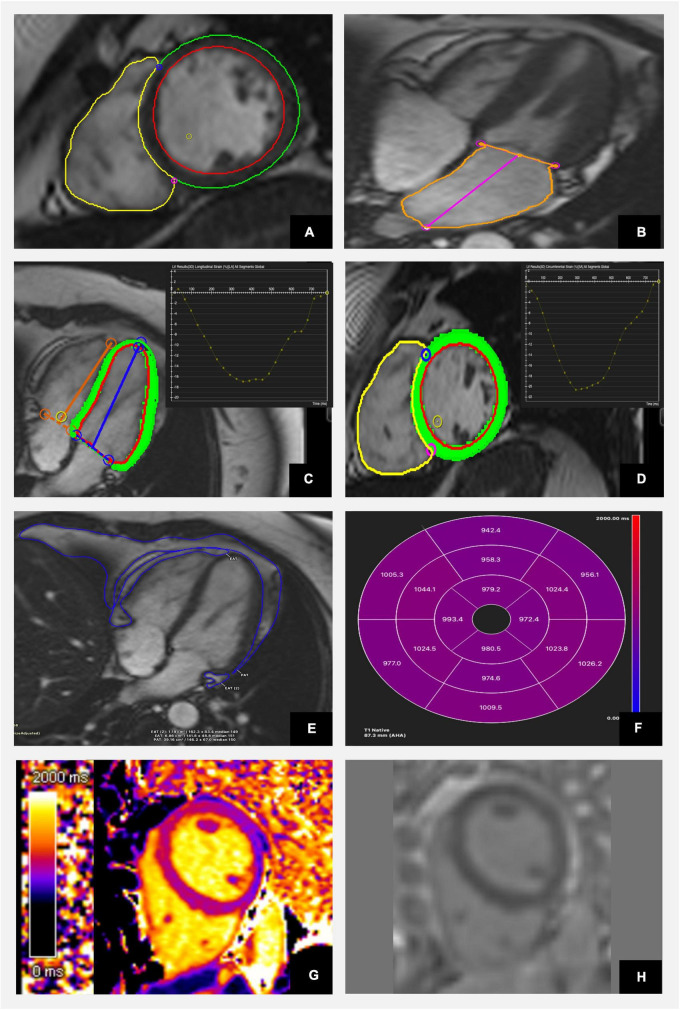
Characterisation of diabetic heart disease with cardiac magnetic resonance including anatomical and functional assessment as well as tissue characterisation using CVI42 software. **(A)** Evaluation of LV and RV volumes and function using short axis stuck images. **(B)** Assessment of left atrial size. **(C)** Analysis of longitudinal and **(D)** circumferential strain. **(E)** Estimation of epi- and pericardial fat tissue and **(F)** 17 segments AHA model representation of average segmental native T1 mapping values. **(G)** Color map of native T1 mapping. **(H)** Late gadolinium images show no evidence of focal fibrosis.

The cardiac consequences of diabetes on the myocardium ensue from metabolic and functional alterations. CMR is a versatile imaging modality (Figure 3) able to describe both ischaemic and non-ischaemic cardiomyopathies and detect haemodynamically significant coronary artery disease. Observational studies described abnormalities in structure and function in patients with diabetes (64, 65). Post mortem studies confirmed distinct ventricular hypertrophy with diffuse fibrotic strands observed between muscle fibres on histopathology and identified it as diabetic cardiomyopathy (66). The strong biological link between diabetes and hypertension and the phenotypic similarity of left ventricular hypertrophy caused by both conditions makes it difficult to distinguish (67). Diabetes was previously described to be associated with left ventricular concentric remodelling and a modestly increased mass (68).

Although cardiac magnetic resonance is mostly used to provide information about systolic function of the left ventricle, it can also characterise diastolic dysfunction and therefore can offer a holistic approach to the early detection of myocardial dysfunction associated with diabetes. Different MRI technique are currently available to evaluate diastolic function (phase contrast imaging for flow analysis, myocardial tagging for regional function analysis) allowing such measurements as longitudinal fractional shortening used when assessing patient presenting with heart failure preserved ejection fraction (HFpEF). Strain-encoded (SENC) (69), displacement encoding with simulated echoes (DENSE) (70) and MRI feature-tracking (MRI-FT) offer additional assessment of the regional function (71). Four-dimensional flow (4D-Flow) MRI is superior to Doppler echocardiography and offers evaluation of intracardiac velocity. Additionally, MRI-derived detection of parameters describing left atrial enlargement and dysfunction have been also shown to be predictive for identifying diastole dysfunction (72). Regrettably, those techniques are time-consuming and require complex post-processing and long image acquisition, what significantly limit its use.

### Myocardial fibrosis

Late gadolinium images confirmed silent myocardial infarctions in 4.3% of asymptomatic type 1 diabetes patients (65) and in 17% of older asymptomatic type 2 diabetes patients (73).

The community-based ICELAND-MI study, which showed that LGE diagnosis of unrecognised myocardial infarction was associated with a 45% increased mortality (74). Novel tissue characterisation techniques such as mapping techniques can demonstrate the degree of interstitial fibrosis observed in patients with diabetes. Extracellular volume (ECV) fraction, a measure of interstitial fibrosis linked to diastolic dysfunction, has been described to be increased in patients with diabetes (75, 76), however, other clinical studies reported inconsistent results regarding the presence of interstitial fibrosis in patients with diabetes (77). Those tissue characteristics by CMR can describe early signs of diabetic cardiomyopathy with the presence of interstitial fibrosis and subclinical abnormalities, which can be also described by tissue tracking images (78). It remains poorly understood if those changes could represent LV remodelling caused by the higher prevalence of hypertension in patients with diabetes.

### Altered metabolism imaged by MRI spectroscopy

MR spectroscopy techniques have been instrumental in describing an increased myocardial triglyceride content in patients with diabetes type 2 (79–81). Myocardial triglycerides can be quantified using hydrogen MRI spectroscopy (^1^H MRS) (82) and has been shown to be increased in ageing, and in patients with diabetes and obesity and is associated with myocardial dysfunction (79, 83). Noteworthy, weight loss was able to partially reverse myocardial triglyceride accumulation in patients with diabetes and therefore improve left ventricular function (81). Future studies will determine if novel glucose-lowering therapies will result in reducing myocardial triglyceride content. Altered myocardial metabolism has been considered among the potential mechanisms leading to diabetic heart disease. The energetic state of myocardium can be measured by phosphorus magnetic resonance spectroscopy (^31^P-MRS), which allows non-invasive assessment of relative concentration of PCr to ATP (PCr/ATP) as a sensitive index (84). Decreased PCr/ATP has been shown to be a predictor of mortality and left ventricle dysfunction (85). Such energetic deficit in diabetic cardiomyopathy is further exacerbated by exercise and is associated with coronary microvascular dysfunction (85). More recently, hyperpolarised ^13^C magnetic resonance spectroscopy has been shown to non-invasively assess physiological and pathological changes in cardiac metabolism in the human heart in health and disease (86). It demonstrated the emerging potential for hyperpolarised imaging in assessing mechanisms underpinning the development of heart failure in diabetes.

### Microvascular dysfunction and the role of stress perfusion MRI

Increased oxidative stress, altered substrate use, and insufficient myocardial perfusion have been proposed as the mechanisms underlying myocardial structural and functional changes observed in the diabetic heart disease. Patients with type 2 diabetes have higher global myocardial perfusion at rest and lower maximal myocardial blood flow during vasodilator-induced stress than control subjects (87). Adenosine stress MRI has been shown to have a good ability to detect haemodynamically significant coronary artery disease in patients with diabetes (88, 89). CMR first-pass perfusion imaging during vasodilatory stress with adenosine or regadenoson has been now widely accepted as the first-choice test for cardiovascular risk stratification of patients with diabetes mellitus. The presence of inducible myocardial ischaemia, defined as at least one positive segment of >1 voxel thickness lasting for at least three heartbeats, was the strongest predictor of clinical outcome (90). However, this is usually evaluated with qualitative or semi-quantitative methods, and it can be inaccurate when myocardial blood flow is globally reduced (in 3-vessel disease). More recently, a dual-sequence protocol was developed to produce an in-line perfusion mapping allowing a pixel-wise quantification of myocardial blood flow (91). This technique can be used to detect physiologically significant CAD, microvascular disfunction and distinguish it from multivessel disease as defined by invasive measurements (92). In a larger cohort of patients with both suspected and known coronary artery disease this AI based approach was shown to be an independent predictor of adverse cardiovascular outcomes (93). Hence, CMR stress perfusion is used to detect highly prevalent multivessel CAD and microvascular angina in patients with diabetes, which is a result of endothelial dysfunction. Automated methods utilising deep learning have been shown to provide one-click analysis and reporting of cardiac perfusion mapping in a manner comparable to manual assessment (94).

### Hybrid cardiac positron emission tomography/magnetic resonance

Over the last decade we have also observed an increased interest in hybrid cardiac PET/MR imaging protocols, which have been incorporated into clinical workflows in many centres. PET is known for its role in quantification of myocardial perfusion and coronary flow reserve as well as visualisation and quantification of metabolic and inflammatory processes at the molecular level (95). Thus, when added to CMR, a technique offering a broad range of capabilities, it is regarded as a promising tool to diagnose and manage metabolic changes leading to myocardial remodelling in diabetic heart disease. A loss in metabolic flexibility reflected in an overdependence on fatty acids as the primary energy source limiting the hearts’ ability to alter substrate metabolism in response to varying physiological and metabolic conditions has been confirmed by preclinical and early human studies using PET/MRI (96). Moving forward a multi-modality imaging protocols interrelating hybrid imaging techniques may be required to improve our understanding of complex processes underlying development of diabetic cardiomyopathy.

## Discussion

**FIGURE 4:**
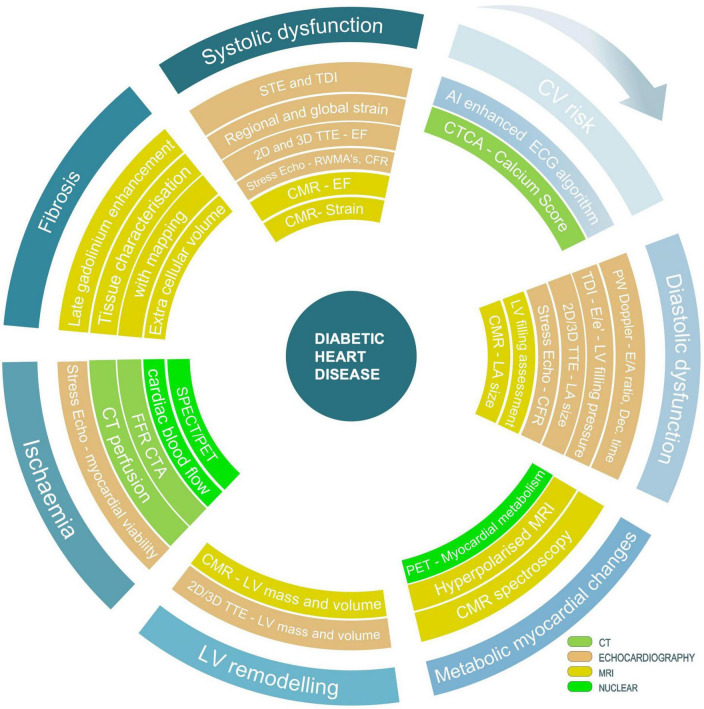
Suggested strategy of using various techniques and imaging modalities in screening patients with diabetes for signs of diabetic heart disease. Outline of currently available techniques and imaging modalities allowing characterisation of different stages of the disease with focus on processes leading to the development of diabetic heart failure.

Although asymptomatic cardiovascular disease is common in people with diabetes and is associated with adverse outcome, the role of multimodality cardiac imaging in screening remains debatable. Large randomised clinical trials testing the value of using imaging in early detection of diabetic heart disease are required to introduce such investigations into clinical practice. The success of novel therapies with proven cardiovascular benefit should motivate such investigations in future and lead to the development of clinical guidance utilising various cardiac imaging modalities in detecting early signs of heart disease in those patients. Patients with diabetes represent a heterogenous group and may require clinical assessment with several imaging modalities in the course of their disease. Figure 4 presents our proposed sequence of using various imaging modalities in screening patients with diabetes. However, several requirements should be met, before a test could be included in a widely accepted screening programme. Firstly, such a test should have superior sensitivity and specificity and be able to differentiate high and low risk patients. Secondly, given high costs of CT and MRI scans the cost-effectiveness of imaging modality should be considered against the benefit of detecting a particular outcome. Thirdly, if screening for early signs of diabetic cardiomyopathy would be considered, it may need to be undertaken in individuals with overall higher risk of HF as defined by other well recognised HF predictors such as more advanced age, hypertension, and microvascular complications of diabetes. This approach would emphasise the role of combining imaging biomarkers with established risk score calculators. We are observing an increasing use of such risk calculators allowing estimation of risk scores of cardiovascular outcomes in patients with diabetes based on their clinical characteristics. UKPDS Risk Engine (97), QDiabetes (98), WATCH-DM (99) and TRS-HF_*DM*_ (100) risk scores are the most frequently cited examples. The advances in using AI algorithms in image interpretation is also very promising. For example, an application of AI to ECG, a routine, widely available, low-cost test has been proved to identify asymptomatic patients with heart failure (101). One could predict that AI- enhanced ECG, which is recorded for each patient with the diagnosis of diabetes could become the first screening tool. It would allow clustering patients into subgroups with differing cardiovascular risks and refining identification of patients requiring appropriate imaging modality to define the stage of their disease.

From the cost-efficiency point of view, echocardiography with strain imaging has the highest chance to be considered as a modality of choice in screening for early signs of diabetic heart disease. Echocardiography remains the most accessible imaging modality and with the use of novel parameters can phenotype various subtypes of heart failure associated with the diagnosis of diabetes. It is also the most versatile technique able to examine and quantify the degree of systolic and diastolic function, provide evidence of regional wall motion abnormalities, assess signs of left ventricular hypertrophy, detect an increased left atrial pressure, and describe the presence of rarer types of inherited and acquired cardiomyopathies.

From the coronary artery point of view, calcium score remains to be a powerful tool for personalised risk assessment and decision making. Patients with diabetes and calcium score of zero have low event rate. Individuals with an increased calcium score may require further assessment with CTA and/or functional test to guide a wide range of preventive therapies and treatment strategies. In symptomatic patients, CTA has a well-established role and the addition of computational fraction flow reserve as well as novel imaging markers such as PCAT may strengthen its role even further. Given the complexity of mechanisms leading to myocardial involvement in patients with diabetes and all strengths and weaknesses of each imaging modality, the role of imaging in screening of asymptomatic patients with diabetes remains questionable.

The core value of cardiac magnetic resonance lies in the tissue characterisation, the detection of diffuse myocardial fibrosis, and an increase myocardial triglyceride accumulation. MR spectroscopy techniques including novel hyperpolarised ^13^C magnetic resonance spectroscopy allow assessment of altered myocardial metabolism in the diabetic heart and holds a promise to uncover the link between diabetes and heart diseases. Noteworthy, MRI provides multiple metrics assessing multi-organ health within one acquisition, which is particularly valuable in case of a systemic disease such as diabetes.

Another way of utilising multi-modality imaging techniques within clinical screening pathways is to focus on the highest risk of certain outcome and choose a test, which is most likely to identify that outcome. For example, in patients with diabetes who are at high risk of developing coronary artery disease, CTA will identify patients with obstructive coronary artery disease or left main stem disease. Depending on local availability, stress echocardiography and perfusion scintigraphy or perfusion CMR could also be considered as first line tests to detect haemodynamically significant coronary artery disease. On the other hand, in those patients who have higher risk of heart failure, ECG and echocardiography will be used as the first line investigations followed by CMR. Figure 4 outlines the strength of each imaging modality and proposes most appropriate use of various techniques.

The growing interest in the use of artificial intelligence in the field of imaging will certainly produce new insights into such complex pathophysiology. The rapid increase in the number of studies confirming superiority of AI algorithms in analysing imaging data is very encouraging. Most importantly, AI will also increase standardisation of interpretation and quantification in imaging. The role of AI has been also confirmed in deep phenotyping of highly heterogenous cohort of patients with diabetes, which on its own could personalise the choice of most appropriate imaging modality in various cohorts of patients. The myriad of applications of AI is a harbinger of a potential breakthrough in how we will come to view the utility of various imaging markers in the early detection of diabetic heart disease is the not-so-distant future.

## Author contributions

MW wrote the first draft of the manuscript and finalised the last version after co-authors comments. MG prepared Table 1 and Figure 4. AR drafted section of the manuscript describing the role of echocardiography. AB prepared Figure 3. All authors contributed to manuscript revision, read, and approved the submitted version.
